# Performance of Void-Free Electrospun SPEEK/Cloisite as a Function of Degree of Dispersion State on Nanocomposite Proton Exchange Membrane for Direct Methanol Fuel Cell Application

**DOI:** 10.3390/membranes9010007

**Published:** 2019-01-02

**Authors:** Nuha Awang, Juhana Jaafar, Ahmad Fauzi Ismail, Mohd Hafiz Dzarfan Othman, Mukhlis A. Rahman

**Affiliations:** 1 Advanced Membrane Technology Research Centre (AMTEC), Universiti Teknologi Malaysia, 81310 UTM Skudai, Johor Bahru, Malaysia; nuhaawang@yahoo.com (N.A.); afauzi@utm.my (A.F.I.); hafiz@petroleum.utm.my (M.H.D.O.); mukhlis@petroleum.utm.my (M.A.R.); 2 Faculty of Engineering, Universiti Teknologi Malaysia, 81310 UTM Skudai, Johor Bahru, Malaysia

**Keywords:** DMFC, void-free membrane, exfoliated, electrospinning, SPEEK/cloisite

## Abstract

One of the main problems in direct methanol fuel cell (DMFC) application is methanol crossover. In order to solve the problem, an exfoliated void-free electrospun Sulfonated Poly(Ether Ether Ketone) (SPEEK)/cloisite nanocomposite membrane was developed. The membrane was prepared by immersing electrospun SPEEK/cloisite fiber mats onto incomplete solidified SPEEK polymer matrix. A well dispersed and reduction size of cloisite particles that ranges from 0.29–0.39 µm was observed by using Scanning Electron Microscopy Analysis (SEM) and Atomic Force Microscope (AFM). The effect of the morphology of the composite membrane in terms of degree of dispersion state of the Cloisite on the membrane performance was discussed. SP/e-spunCL15 with fully exfoliated structure exhibited the highest performance as compared to other tested membranes and Nafion^®^ 115 with current density of 1042.2 mAcm^−2^ and power density of 1.18 mWcm^−2^. Improved morphological, dimensional change properties, and performance assigned to well-dispersed cloisite15A induced by the electrospinning technique make the membranes more efficient for direct methanol fuel cell applications.

## 1. Introduction

Nowadays, the direct methanol fuel cell (DMFC) has become one of the most favorable power supplies for portable devices. The advantage of methanol as energy carrier are easy to transport and handle, easy to directly oxidized to carbon dioxide and water, less hazardous than hydrogen since the concentration that generally applied in DMFC is low (typically 1 Molar). Besides this, methanol can be produced from biodegradable sources and various natural sources for example wood, coal, and gas [1,2].

The most common type of polymer that applied as polymer exchange membrane (PEM) is Nafion^®^ from DuPont. Although Nafion^®^ owns good proton conductivity, highly hydrophobic of perfluorinated backbone and highly hydrophilic of sulfonic acid functional group in Nafion^®^ has limited the performance of Nafion^®^ as PEM [3,4]. The adverse effect can be obviously observed in the presence of water due to nano separation or the aggregation of hydrophilic domain which water and methanol are both transported across Nafion^®^. The phenomenon is called methanol crossover. Methanol crossover from anode to cathode is detrimental to performance of DMFC.

Recent studies have proved that the incorporation of clay type inorganic fillers into non-fluorinated polymer, such as Sulfonated Poly(Ether Ether Ketone) (SPEEK), can reduce methanol crossover, reducing swelling without reduction of proton conductivity by the expansion of interspacing layers. Despite all the improvements, poor uniformity dispersion of inorganic clay layers in SPEEK had become the limitation in developing a morphologically good PEM for DMFC application [4,5,6,7].

From morphological point of view, an exfoliated or delaminated structure is a promising interfacial structure for a high performance nanocomposite. Since exfoliated structure is often related with good dispersion of fillers in nanocomposite, electrospinning could be one of the promising fabrication techniques to produce good dispersion fillers: conductive, fibrous, and a high specific area [8,9,10,11,12].

Recently, there are a number of processing techniques that have been used to fabricate PEM for example template synthesis, drawing, self-assembly phase separation, and electrospinning. Electrospinning is known as an efficient fabrication method for polymer nanofibers production. In 2010, it was found that polymers mostly in solution and some in melt form have been successfully spun into ultrafine fibers which potentially been applied as reinforcement in nanocomposite development [10]. Based on the discovery, the electrospinning is seemingly a promising method for mass production of one-by-one continuous nanofibers from numerous polymers.

Unfortunately, electrospinning produces highly porous membranes of fibrous mat. Thus, a finishing method needs to be studied in producing a dense membrane for DMFC application. Apparently, studies on impregnation of electrospun SPEEK-cloisite fibers on SPEEK matrix as PEM for DMFC application have not been reported. Therefore, the study aims to prepare a novel void-free electrospun SPEEK/cloisite nanocomposite. According to the features proposed from the previous studies, this type of fabrication aimed to produce an exfoliated morphological membrane with high performance PEM for DMFC application.

## 2. Experimental

### 2.1. Materials

The main material which acted as polymer matrix, the powder type of Poly(ether ketone) (PEEK) bought from Victrex Inc., Victrex Technology Centre Hillhouse International Thornton Cleveleys Lancashire FY5 4QD, UK. The fillers was Cloisite 15A^®^, purchased from Southern Clay Products, Inc., Texas, USA. 95–97% purity of sulfuric acid (H_2_SO_4_) (sulfonating agent) was acquired from QRec (Asia), Rawang, Selangor, Malaysia. The solvent utilized in the analysis was Dimethylsulfoxide (DMSO) and it was acquired from Sigma Aldrich (M) Sdn. Bhd. Petaling jaya, Selangor, Malaysia. Nafion 115^®^ was purchased from Sigma Aldrich (M) Sdn. Bhd. Petaling jaya, Selangor, Malaysia.

### 2.2. Synthesis of Sulfonated Poly(Ether Ether Ketone) (SPEEK)

The sulfonation was carried out to give SPEEK with degree of sulfonation 64%. The test was done by referring to the previous investigation [12]. 50 g of SPEEK powder was mixed with 1000 mL of H_2_SO_4_ completely for 1 h at room temperature. The temperature was raised to 50 °C for 3 h. The precipitated of the SPEEK solution was poured into ice water basin. The SPEEK was sifted and washed consistently with deionized water until the point when the pH of the deionized water changed to 6–7. The SPEEK then dried in vacuum oven at 80 °C for 24 h.

### 2.3. Preparation of Electrospinning Dope Solution

Desired amounts of Cloisite 15A^®^ (0.10 wt.%, 0.15 wt.%, 0.20 wt.%, 0.20 wt.%, and 0.30 wt.%) were stirred in DMSO at 60 °C for two hours before adding to SPEEK with 10 wt.% which was dissolved earlier in DMSO. Each formulation was vigorously stirred for 24 h at 60 °C to create a homogeneous solution.

### 2.4. Electrospinning of Nanofibers

A high voltage control (NF1000 ES93053) was connected to the Cloisite dope solution. The dope was sent to the needle tip through syringe pump with flow rate of 1 mL per hour to control the arrangement stream rate. Electrospun fibers were acquired on an electrically grounded aluminum foil that put vertically to the needle tip. The distance between needle tips to screen collector was set at 18 cm with voltage applied of 22.25 kV.

### 2.5. Preparation of Void-Free SP/e-spun Cloisite Membrane

**Figure 1:**
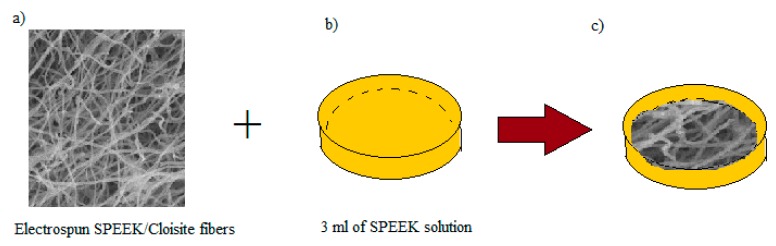
(**a**–**c**) The preparation steps in producing void free dense SP/e-spunCL membranes [1].

6 mL of SPEEK/Cloisite solution was electrospun (with syringe pump rate of 1 mL per hour) as indicated by the parameters chose (See Figure 1a–c). In the interim, the SPEEK solution was filled a petri dish as a base for the layer as represented in Figure 1b) and left for 4 h for incomplete solidification process. The electrospun fiber was placed onto the incomplete solidified SPEEK as appeared in Figure 1c). The membrane was left in petri dish for 24 h at room temperature before drying process in the oven for an additional 24 h at 60 °C. The layer was cast from petri dish at that point washed by H_2_SO_4_ for cross linking process. The membrane was then washed again with water and dried 24 h at 60 °C. Table 1 outlines the naming of the electrospun fibers and membrane samples.

### 2.6. Characterization

#### 2.6.1. Atomic Force Microscope (AFM)

The exfoliated structure had been proven by AFM test handled at Universiti Tun Hussein Onn Malaysia (UTHM) using XE100 AFM machine from Park System, Suwon, Korea. Tapping mode applied with constant amplitude and approximately 300 kHz of resonance. The image recorded with scan rate of 2 Hz with resolution of 512 samples per line.

#### 2.6.2. Fourier Transform Infrared Spectroscopy

FTIR spectrometer (SHIMADZU, IRT RACER-100, Osaka, Japan) was performed to study the chemical structure of organic molecules and potential structural changes that occurred as a result of the membrane physical treatment or degradation and to observe structural changes prior electrospinning process. The SP/e-spunCL membrane and cast SPEEK/cloisite membrane were put inside a diamond cell for the FTIR spectra recording. The spectra were normally recorded in absorbance mode over a wave number range of 4000 to 700 cm^−1^.

#### 2.6.3. Scanning Electron Microscopy Analysis (SEM)

The morphology of SP/e-spunCL membranes and e-spunCLs, Energy-dispersive X-ray spectroscopy (EDX) together with surface mapping for high magnification was observed using scanning electron microscopy (SEM) (HITACHI TM3030, Osaka, Japan). The confidence percentage of analysis is 98%. Samples were prepared by freezing the dry membrane samples in liquid nitrogen and breaking them to produce a cross-section (for membrane samples). The samples were later put through a vacuum spattered with a thin layer of gold before SEM examination. 

#### 2.6.4. Field Emission Scanning Electron Microscopy (FESEM)

The morphology behavior of SP/e-spunCLs had been further investigated using field emission scanning electron microscope (FESEM) JEOL JSM-7600F, JEOL USA Inc., Peabody, MA, USA. All membrane samples were frozen, dried, and broke to produce a cross-section. Fresh cross-sectional of the membranes were vacuum spattered with a thin layer of gold before FESEM examination to examine the existence of electrospun fibers which could give the significant feature on the cross section of the membrane.

#### 2.6.5. X-ray Diffraction Analysis

XRD is an analysis method that is applied to identify amorphous and crystallinity of cloisite in membrane matrix. The test is important as preliminary observation to define the exfoliation surface of membranes. The analysis used XRD (Philip PW1710 XRD, Fonteinkruid 134617 JE Bergen op Zoom, Philips Analytical, North Brabanth, The Netherland) with nickel-filtered Cu-Kα source (*λ* = 0.154056 nm) at 30 kV and 30 mA. The diffractograms were scanned with a scanning rate of 2° min^−1^ in a 2*θ* range of 1.5° to 10° at a room temperature. The *d*-spacing of Cloisite15A^®^ itself and in all SPEEK nanocomposites prepared was calculated from Bragg’s equation using XRD results:

(1) $$d=\frac{n\lambda }{2\sin\theta }$$

where *d* is the spacing between layers of the clay, *λ* the wave length of X-ray equal to 0.154056 nm, *θ* the angle at the maximum point of the first peak (lowest *θ*) in the spectra and *n* is a whole number, representing the order of diffraction, *n* = 1 in the calculation. 

#### 2.6.6. Dimensional Stability

The dimensional stability of membrane samples in plane and thickness were observed similar to the water uptake test were implemented. Nevertheless, the dimensional change of membranes was calculated using Equations (1) and (2). 

For the dimensional change ratio in thickness, the thickness of the wet tested membranes (SP/e-spunCL10, SP/e-spunCL15, SP/e-spunCL20, SP/e-spunCL25, and SP/e-spunCL30) was measured by using the digital micrometer and three replicates data was taken and the results were presented as average data.

(2) $$\mathrm{Dimensional}\;\mathrm{change}\;\mathrm{ratio}\;\left(\mathrm{plane}\right)=\frac{L_{wet}-L_{dry}}{L_{dry}}\times 100\%$$

(3) $$\mathrm{Dimensional}\;\mathrm{change}\;\mathrm{ratio}\;\left(\mathrm{thickness}\right)=\frac{d_{wet}-d_{dry}}{d_{dry}}\times 100\%$$

where, *L_wet_* is the length of the wet membrane and *L_dry_* the length of the dry membrane, whereas *d_wet_* is the thickness of the wet membrane and *d_dry_* the thickness of the dry membrane.

#### 2.6.7. Dissolution Test and Water Uptake

The stability test of membranes was then continued by extending the duration of membranes in water at room temperature. The study also called dissolution test. The membranes were soaked in water for as long as the thickness as well as planed able to be sustained dimensionally.

Water uptake was studied to observe swelling behavior of membranes. The different of membranes weight (wet and dry) were observed. The observation is critical in order to find the hydration sufficiency as well as mechanical strength in performing as PEM for DMFC system. The membranes were dried for 48 h in an oven at 60 °C were weighed. The membranes with diameter of approximately 5.0 cm were soaked in deionized water overnight at room temperature and blotted dry with absorbent paper were reweighed. The water uptake was calculated as follows:

Water uptake = *G_w_* − *G_d_*/*G_d_* × 100% (4)

where, *G_w_* is the weight of the wet membranes and *G_d_* the weight of the dry membranes.

### 2.7. Single PEM Direct Methanol Fuel Cell Test

#### 2.7.1. Preparation of Membrane Electrode Assembly (MEA)

The active surface area of the MEA was 4.0 cm^2^ and comprised of PtRu-supported carbon catalyst (1.0 mg/cm^2^ as Pt amount). MEAs from SP/e-spunCL10, SP/e-spunCL15, SP/e-spunCL20, SP/e-spunCL25, SP/e-spunCL30 and Nafion115^®^ membranes were prepared by hot pressing the membranes on the electrodes with 19 Nm pressure and 40 °C for 1 min. 

#### 2.7.2. Single DMFC Performance Testing

**Figure 2:**
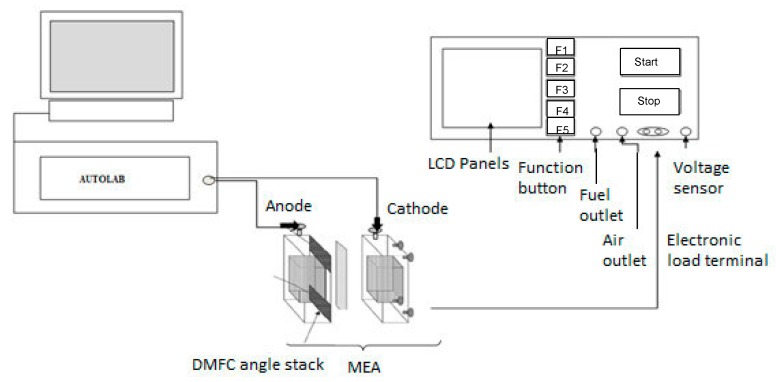
The schematic diagram of fuel cell analyzer test system. DMFC: direct methanol fuel cell; MEA: Membrane Electrode Assembly.

The single DMFC performance was evaluated by recording the cell voltage vs. current density curves using a fuel cell analyzer test system (AUTOLAB Metro hm B.V, Fonteinkruid 134617 JE Bergen op Zoom, The Netherland), see Figure 2. The electronic loads used in these experiments have a combined maximum current of 240 A, and a voltage range of 0–60 V. AUTOLAB provides NOVA 1.5 software to measure Voltage–Current performance of DMFC.

The method utilized in the NOVA programming was intended to quantify the capability of the stack during 30 s, utilizing an interval time of 10 milli second, for each estimation of the current drawn from the stack. The estimation of the release current was directly controlled by the software. The values changed in the range of 0 and 1 A.

The gold coated electronic load terminal was designed to exactly measure load by minimizing electric resistance. The current load, temperature, methanol flow rate at 1 mL/min, air and fuel flow rate constant at 100 mL/min can varies automatically. The current loadings were varied from 50–300 mAcm^−2^ for 20 min. The sequence current changes were repeated two times after cell temperature became 60 °C. Once the temperature became 60 °C, the DMFC performance measurement was conducted three times, and the results were presented as the average data. The polarization curves were obtained using an Autolab PGSTAT302N in combination with the 1.11.0 NOVA software.

## 3. Results and Discussion

### 3.1. Structural Characterization of SP/e-spunCL Membranes

**Figure 3:**
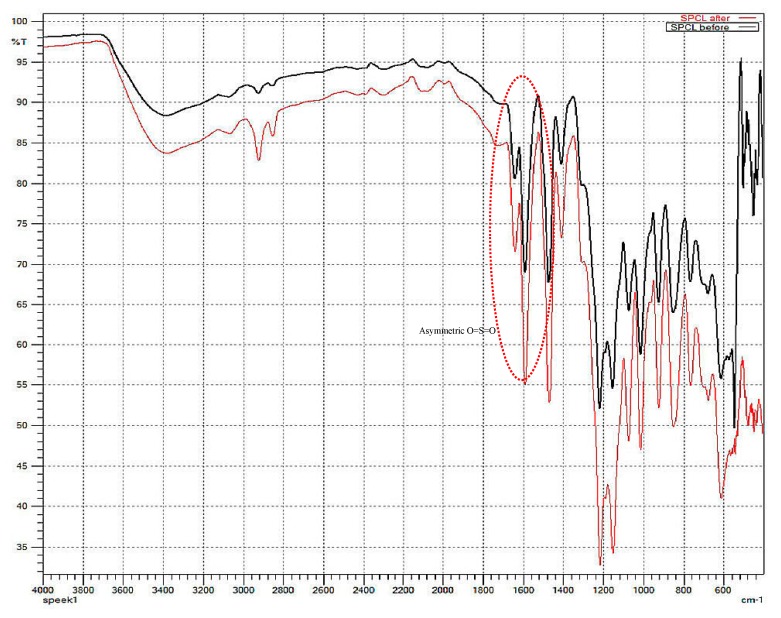
FTIR spectra on casted Sulfonated Poly(Ether Ether Ketone) (SPEEK)/Cloisite membrane and SP/e-spunCL membrane.

In order to confirm that the applied high electrical field does not cause any change in the chemical structure of SP/e-spunCL membranes, the FTIR spectra of a cast SPEEK/Cloisite membrane and SP/e-spunCL membranes are compared (Figure 3). The two spectra are identical showing the presence of characteristic peak of 1600 cm^−1^–600 cm^−1^ [11]. The details of transmission bands are shown in Table 2.

Since it is crucially vital to examine the morphological types (intercalated and exfoliated) alongside the effects of the two highlights on the membrane properties, surface structure of the membranes were investigated with three basic strategies which are Scanning electron microscopy (SEM), X-Ray diffraction (XRD) and Field Emission Scanning Electron Microscope (FESEM).

**Figure 4:**
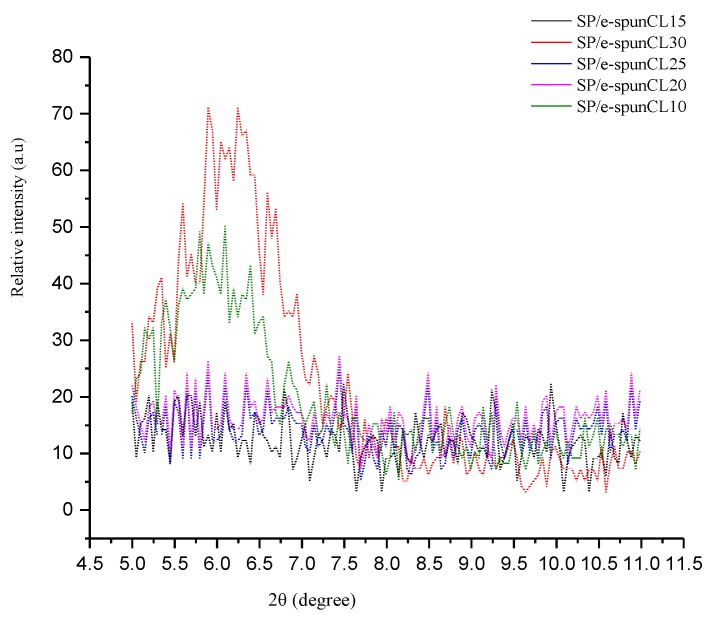
XRD of all SP/e-spunCL membranes.

XRD investigation was used in the research to look at the microstructure and portray the clay scattering with SP/e-spunCL layers. Note that the presence of organic materials (SPEEK) lead the featureless peak happen at the typical pinnacle that unadulterated closite 15A was regularly seen at 2*θ* = 2.6° [11]. Thus, the examples of SP/e-spunCL layers were seen at 2*θ* = 5°. The XRD patterns of the corresponding clay in Figure 4 were only observed in a small angle part in the range of 2° to 10° of 2*θ* scale because the dispersion state of the clay particles can simply be obtained by analyzing the (001) lattice spacing of the clay.

Referring to the Bragg’s law equation, the expanding of d-spacing causes the widening and shifting of the XRD patterns. The peaks likewise characterized the morphology as intercalated. From the two peaks, it very well may be seen that SP/e-spunCL10 demonstrated lower angle when contrasted with SP/e-spunCL30. This can be clarified that the layer spacing was expanded because of exfoliation or intercalation effects [12,13,14]. The other membranes which are SP/e-spunCL15, SP/e-spunCL20 and SP/e-spunCL25 demonstrated no peak showed up [9]. The result demonstrated that cloisite were effectively dispersed in polymer matrix because of the electro spinning procedure and the hydrophilicity of cloisite [15]. The missing peak of the three membrane samples can also be induced that the completely exfoliated structure obtained from the introduction of electro spun fibers of SPEEK/cloisite with 15 wt.%–25 wt.% of cloisite loadings into SPEEK polymer matrix.

Excluding SP/e-spunCL10 and SP/e-spunCL30 membranes, the intensity of the peaks that corresponds to the plane (001) increased as the loading of cloisite was below 0.15 wt.% and above 0.25 wt.%. The high intensity of the corresponding peak for the SP/e-spunCL10 and SP/e-spunCL30 membranes occurred probably because of lack of cloisite dispersion on the surface of the membranes as confirmed by the EDX images. SP/e-spunCL10 and SP/e-spunCL30 membranes showed the diffraction peak at 2*θ* = 5.8° (*d*_001_ = 1.52 nm) and 6.10° (*d*_001_ = 1.45 nm), respectively. Since that both particular composite membranes showed higher *d*_001_ than the pure cloisite membrane (2*θ* = 7.1° (*d*_001_ = 1.25 nm) [15,16], it means that the intercalated nanocomposite is obtained.

Even though XRD is a good conventional method that offers a technique which quantitatively analyzes morphology of the membranes, subsequently, another qualitative investigation to finish the morphological examination was conducted to support the existing results. The methods were checking electron microscopy (SEM) and Field Emission Scanning Electron Microscope (FESEM).

The methods not only provided energy dispersive X-ray (EDX) in determining the presence of important element which was cloisite in SP/e-spunCL membrane but also the exfoliation images of surface membranes. Although the methods only provided the information on a small area which was not representative of the overall microstructure, regardless of all, SEM, FESEM, and XRD are very crucial analyses in evaluating morphology of nanocomposites [17].

**Figure 5:**
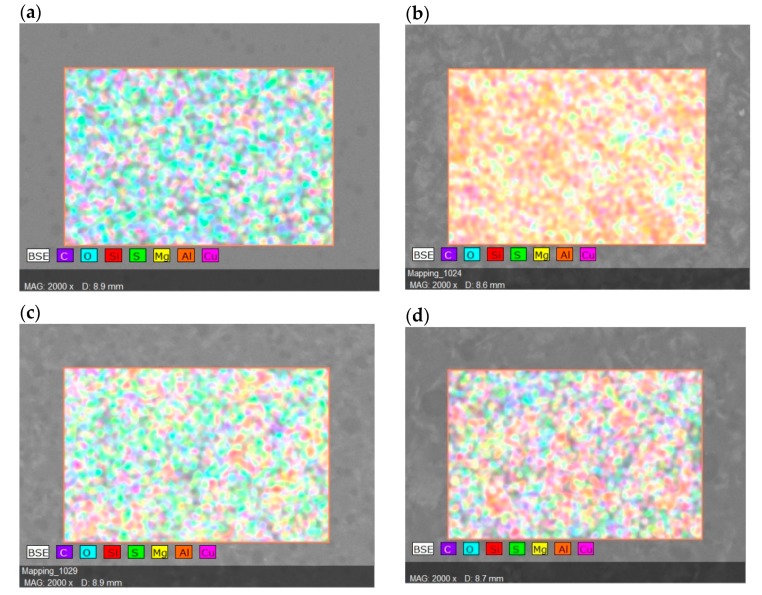

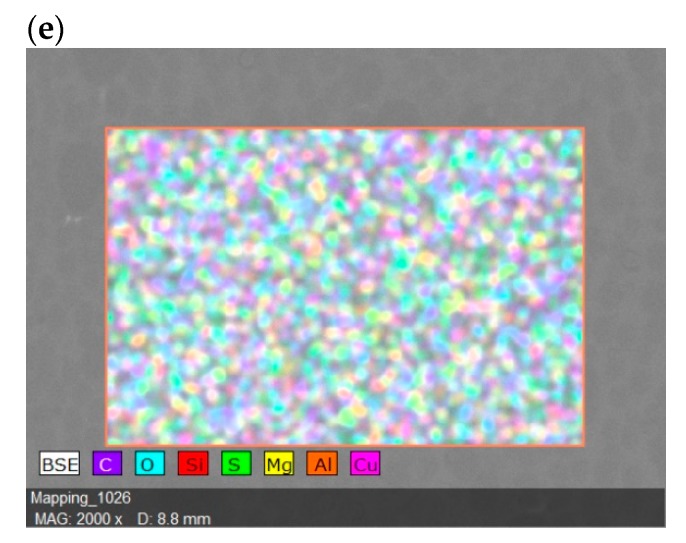
EDX qualitative analysis of cloisite (Si) distribution in SP/e-spunCL nanocomposite membranes (**a**) SP/e-spunCL10, (**b**) SP/e-spunCL15, (**c**) SP/e-spunCL20, (**d**) SP/e-spunCL25, (**e**) SP/e-spunCL30.

Energy dispersive X-ray (EDX) was applied to observe the elements that presented in membranes together with the dispersion percentage of the desired element which was cloisite. The highest percentage of cloisite dispersion was found in SP/e-spunCL15 (13.89%) followed by SP/e-spunCL25 (13.36%), SP/e-spunCL20 (11.36%), SP/e-spunCL10 (11.29%), and SP/e-spunCL30 (11.23%) respectively. The effect of dispersion can clearly observed quantitatively and qualitatively by SEM and FESEM images (Table 3 and Figure 5).

**Figure 6:**
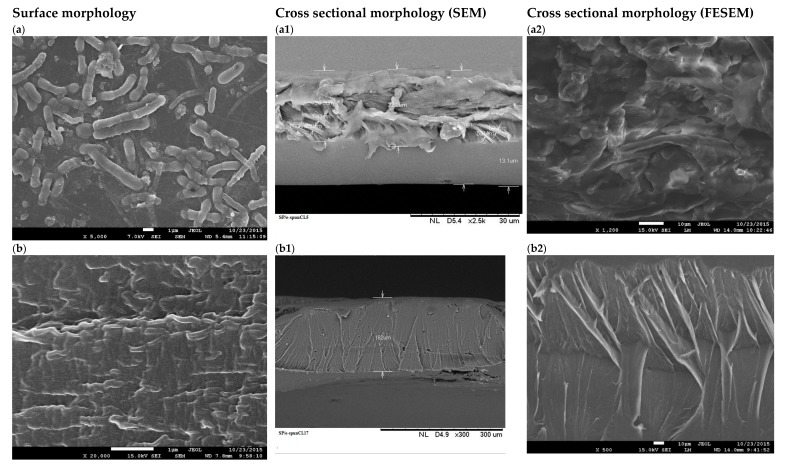

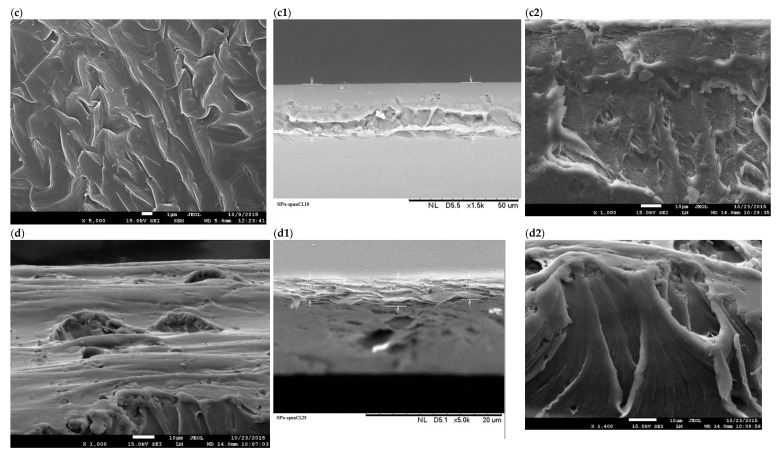

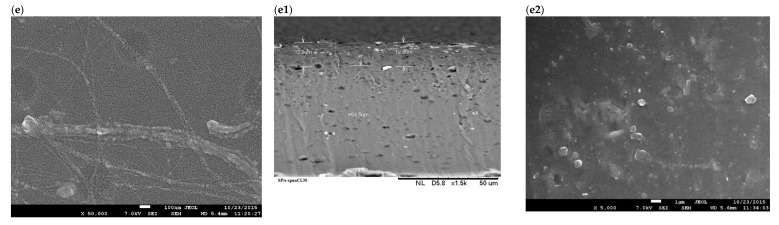
(**a**–**e2**): (**a**–**e**) FESEM surface images of all membranes (**a**) SP/e-spunCL10, (**b**) SP/e-spunCL15, (**c**) SP/e-spunCL20, (**d**) SP/e-spunCL25, (**e**) SP/e-spunCL30), (**a1**–**e1**) SEM images of SP/e-spunCL cross sectional areas (**a1**) SP/e-spunCL10, (**b1**) SP/e-spunCL15, (**c1**) SP/e-spunCL20, (**d1**) SP/e-spunCL25, (**e1**) SP/e-spunCL30), (**a2**–**e2**) FESEM images of SP/e-spunCL cross sectional areas (**a2**) SP/e-spunCL10, (**b2**) SP/e-spunCL15, (**c2**) SP/e-spunCL20, (**d2**) SP/e-spunCL25, (**6e2**) SP/e-spunCL30).

To further the investigation on the exfoliation of SP/e-spunCL membranes, FESEM analysis with few thousand magnifications was carried out. The FESEM and SEM surface and cross sectional images of all SP/e-spunCL membranes were presented in Figure 6(a–e2).

SP/e-spunCL15, SP/e-spunCL20 and SP/e-spunCL25 showed the fully exfoliation structures occurred on the both surface and cross section which were agreed with XRD result. All samples showed crumpled and fibrous patterns on the surfaces which resulted from the addition of electrospun fibers on the SPEEK matrix. As for the cross section images, all three samples had proven the electrospun fibers did not only exist on the surface but also inside the membranes. However the fibrous structures that formed did consistently be found in the whole section of cross sectional areas or it can be found more at the surface rather than the bottom part. The structure can be related as sewing pattern like structures.

SP/-spunCL10 which more exfoliated compared to SP/e-spunCL 30 as defined by XRD also agreed with result in FESEM images. SP/e-spunCL10 showed the existence of cylindrical bacterial like structures on the surface with huge agglomerated sectional areas which SP/e-spunCL30 showed lowest existence of neither fibrous structure nor cylindrical structure. Only few long threads like structures can be observed on the SP/e-spunCL30 surface. From the observation, the different of morphological structures can be explained that the concentration of dope solution with specific electro spinning setting played important role in governing the patterns and performance of membranes.

Although many researchers observed different dispersion of cloisite was proven to give the different impacts on membrane morphologies [18,19,20,21,22,23,24], the study on size of cloisite together with volume of water that can be retained by the particle is scarce. The observation of closite size reduction as a result of electrospinning was done by using AFM.

### 3.2. The Effects of Morphological Structures and Closite Dispersion on Barrier Property of Void-Free SP/e-spunCL

**Figure 7:**
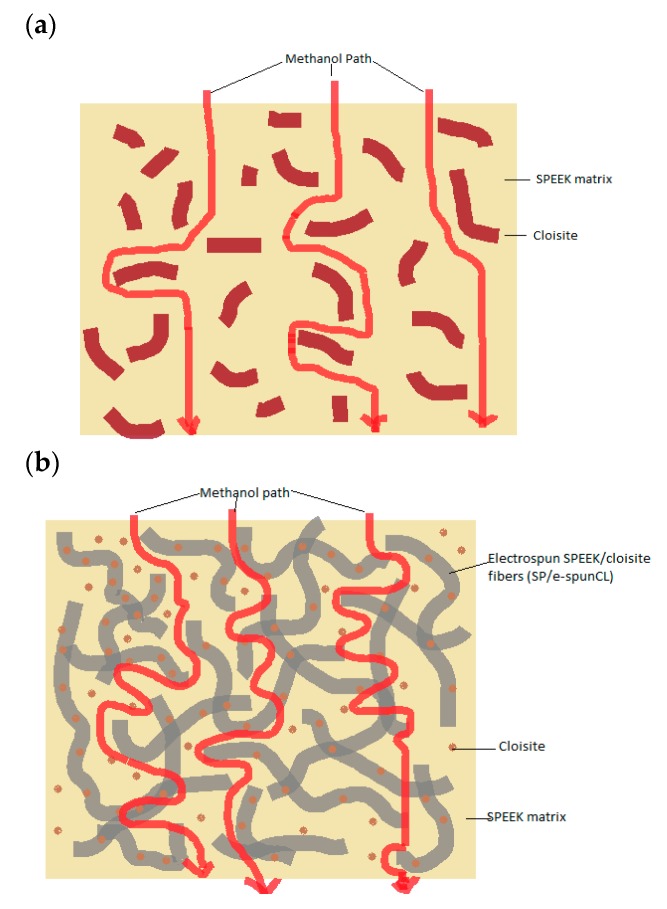
The comparison of methanol path between (**a**) SPEEK/cloisite membranes (**b**) Void-free SP/e-spunCL.

The dramatic improvements in barrier properties were observed as the impacts of addition of electrospun fibers into SPEEK polymer matrix. Clay sheets are regularly impermeable and the existence of clay helps to extend the barrier properties of polymers by making an all the more winding way that retards the dissemination of gas molecules through the polymer matrix (Figure 6) [25,26]. Figure 7 showed the distinction of methanol path between SPEEK/cloisite membrane and SP/e-spunCL membrane.

The improvements of barrier properties rely upon the level of tortuosity made by cloisite layers in the molecules path way in polymeric membranes [27,28]. The tortuous path is controlled by the proportion of real separation which diffusive molecules (methanol and water) are transferred to the most limited separation to diffuse (thickness of membranes). Previous study reported that the barrier properties of polymer/clay nanocomposites against the dissemination of gases and vapors [29,30,31,32]. This was because of the dispersion of clay in the polymeric matrix. Other than this, the exfoliation factor and degree of dispersion cause to the more barrier improvement in the polymer matrix.

Aside from creating more winding path and exfoliated structure to a membrane, another addition essential study on the effect of electrospun fibers on membrane morphology is the reduction of the cloisite size and volume of water that can be retained by cloisite. The retained water can be greatly effect proton conductivity as well as performance of membranes as PEMs in DMFC application. Table 4 presents the summary of information obtained from 10 µm^2^ of AFM images.

As compared to the original size of commercialized cloisite 15A (2–13 µm) [33], after electro spinning applied to the SPEEK/cloisite solution, the sizes of the particle had been reduced to 0.286–0.390 µm. This had proven the electro spinning is the effective method for inorganic particle size reduction. The number of cloisite as well as volume and water retained of particle agreed with the dispersion trend in Table 4 which showed the highest for SP/e-spunCL15 (1668 with volume of 2.914 × 10^−3^ µm^3^, water retained of 4.854 µm^3^ per 10 µm^2^) and the lowest for SP/e-spunCL 30 (1097 with volume of 4.505 × 10^−4^ µm^3^, water retained of 0.494 µm^3^ per 10 µm^2^). The existence of cloisite as fillers had improve the proton conductivity of membrane by the ability of retaining water which is a proton transport medium [33].

### 3.3. Dimensional Stability of SP/e-spunCL Membranes

Studies revealed large interfacial area in well dispersed nanocomposite improved modulus property [34]. Therefore, one of the main reasons in incorporating cloisite to SPEEK polymer is to improve mechanical property. In this study, electro spinning was observed to produce smaller particles and better dispersion. These characteristics enhanced interfacial contact with better dimensional stability. Table 5 showed swelling property and dimensional changes of all SP/e-spunCLs samples after immersing in water.

A good PEM required a good swelling in thickness direction [35]. This is due to the elongation in thickness behavior contributes to the contact between current collectors and membrane electrolyte assembly (MEA), thus increase the overall DMFC performance [35,36]. From the result, the electro spinning method which resulted exfoliated structure membrane together with smaller cloisite particle size with good dispersion significantly contributed to a good impact on swelling behavior of SP/e-spunCL membranes. It can be observed that the addition of electrospun SPEEK/cloisite fibers in SPEEK matrix decreased the swelling ratio in plane direction and increased that in thickness direction.

### 3.4. Long Term Stability of SP/e-spunCL Membranes in Hydrated State

Proton conductivity, mechanical properties, and barrier properties are affected by amount of absorbed water [37]. Thus, study of long-term stability of membrane in hydrated state is crucial for DMFC application. A good PEM requires a dimensionally stable membrane without high percentage of swelling. Swelling caused by excessive water absorbed by hydrophilic group in sulfonic acid group presence in membrane. The excessive absorption contributes on morphological instability, mechanical fragility, and dimensional instability [38].

**Figure 8:**
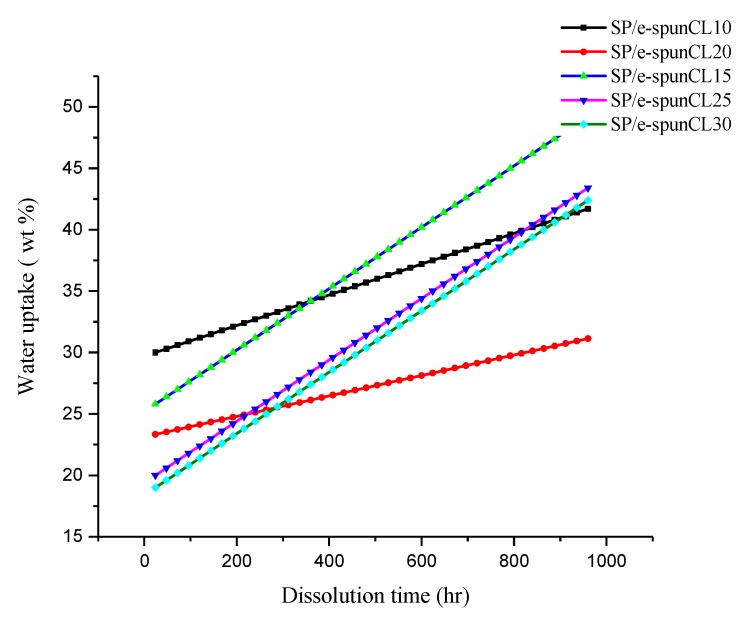
Long term stability effect in hydrated conditions of SP/e-spunCL nanocomposite membranes.

**Figure 9:**
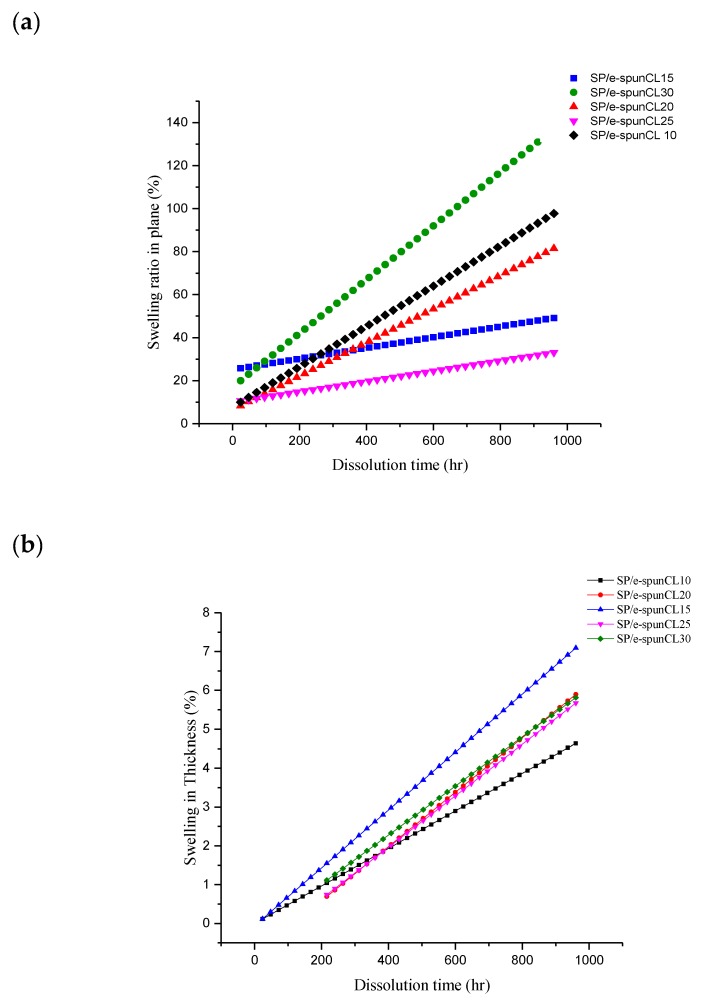
Swelling ratios in (**a**) plane and (**b**) thickness of SP/e-spunCL nanocomposite membranes.

Besides swelling, dimensional change affects performance of PEMs. The high dimensional change in plane is not required for good PEMs due to a weak contact between catalyst and electrolyte membrane [39]. Figure 8 showed the water uptake and dissolution time for SP/e-spunCL membranes, while Figure 9a,b demonstrated the swelling ratio of SP/e-spunCL membranes in the directions of plane and thickness.

From Figure 8, the water uptake of SP/e-spunCL15 was the highest throughout the experimental period among the five tested membranes. It can be suggested that the less-uniform distribution of Cloisite15A^®^ particles in SP/e-spunCL10, SP/e-spunCL20, SP/e-spunCL25, and SP/e-spunCL30 (see Figure 5) allowed the sulfonic acid groups in the SPEEK polymer matrix to absorb water. This is because when a large number of O atoms in sulfonic acid groups are left unattached to Cloisite15A^®^ via hydrogen bonding, a large amount of water will be taken into the membrane via bonding between the O atoms in sulfonic groups and hydrogen atoms in water [40]. 

The swelling data reported in Figure 9b showed similar trend to water uptake. The water uptake and dimensional change in thickness of SP/e-spunCL15 was observed the highest among other membranes. However, SP/e-spunCL15 showed the smallest value for dimensional change in plane which is 25% (Figure 9a). This study indicates that SP/e-spunCL10, SP/e-spunCL20, SP/e-spunCL25, and SP/e-spunCL30 membrane became more fragile with time and mechanically less stable in water than the SP/e-spunCL15 membrane However, all membranes had shown increment dimensionally as well as plane throughout the test period. This indicated that, the free volume for water adsorption as well as the mobility of polymer chains increased with time, which contributed to the increment of water absorbability of membranes. All membranes presented a much higher swelling ratio in thickness than in plane. Similar observation was reported by Juhana 2011 [41].

A PEM with water uptake less than 50% is considered stable and low degree of swelling [42]. Hence, it can be concluded that the swelling effect on SP/e-spunCL15 and SP/e-spunCL25 can be considered as low degree of swelling. Thus, SP/e-spunCL15 and SP/e-spunCL25 membrane is stable enough to be applied in the real DMFC application in hydrated state.

### 3.5. DMFC Performance

**Figure 10:**
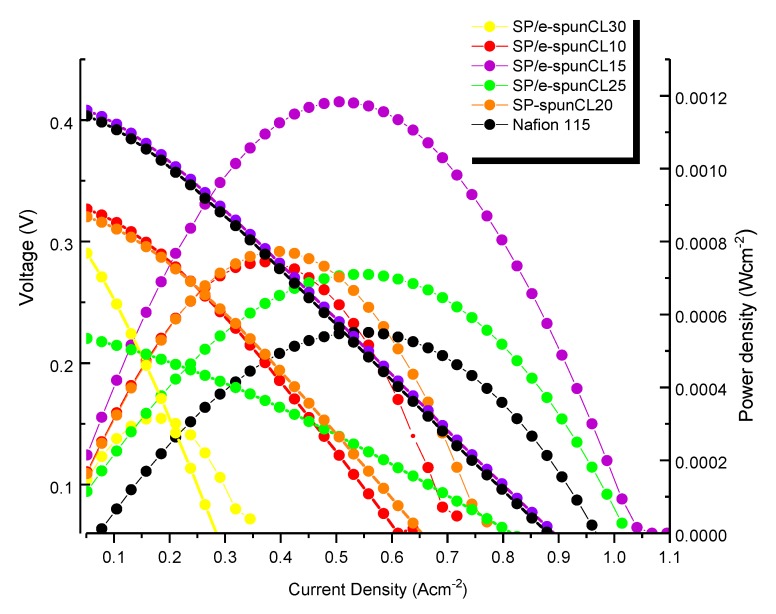
Polarization curve of cell voltage and power density versus current density of SP/e-spunCL membranes and Nafion^®^115 membranes.

The impact of methanol permeability and proton conductivity were verified by polarization measurement [43]. Figure 10 presented the cell voltages as a function of current density for the single cell of DMFC prepared from SP/e-spunCL membranes along with the performance of and Nafion^®^115 membranes for comparison. It was observed that SP/e-spunCL15 exhibited highest performance as compared to other SP/e-spunCL membranes and Nafion^®^115 membranes. Hence, it can be concluded that the performance of the DMFC was improved by exfoliated structure of membranes.

The maximum current densities of SP/e-spunCL15, SP/e-spunCL25, SP/e-spunCL20, SP/e-spunCL10 and SP/e-spunCL30 membranes measured are 1.0422 Acm^−2^, 1.0132 Acm^−2^, 0.7773 Acm^−2^, 0.7216 Acm^−2^ and 0.3472 Acm^−2^, respectively, and 0.9615 Acm^−2^ for Nafion^®^115 as a comparison. The lower current densities of the SP/e-spunCL10 and SP/e-spunCL30 membranes were due to the lower proton conductivity of those membranes than that of other SP/e-spunCL nanocomposite membranes. Lower proton conductivity attributes in obvious ohmic losses causes by high membrane resistance and shows mass transfer limitations [44].

The open circuit voltage (OCV) for SP/e-spunCL15, SP/e-spunCL25, SP/e-spunCL20, SP/e-spunCL10, and SP/e-spunCL30 membranes measured are 0.412 V, 0.223 V, 0.323 V, 0.330 V, and 0.289 V, respectively, and 0.403 V for Nafion^®^115. OCV defines methanol permeation and it increases when the methanol crossover is decreased. Methanol crossover avoids oxygen reduction at the anode and consequently causes to a drastic decrease in the OCV [45]. SP/e-spunCL15 showed the highest methanol barrier property (as presented in Table 4), it reduced the possibility for the methanol to transport towards the opposite electrode thus resulted in higher OCV.

The OCV result especially for Nafion^®^ 115 membrane found a low value which can be related not only caused by methanol crossover. As compared to other comparable materials and fabrications in Table 5, it can be deduced that MEA set up also plays important role in determining a high OCV value. Hence, the inappropriate MEA set up was a reason of the low OCV value of Nafion^®^ 115 membrane. The incompatibility between the membrane and electrode caused methanol leakage directly from anode to cathode without passing through the electrolyte membrane first [46]. Table 6 presented the OCV value together with current and power density of newly developed SP/e-spunCL 15 compared to other researches. It showed that the newly developed membrane has a potential as a high performance PEM in the future due to the highest current density presented.

Figure 10 presented the polarization curve of power densities as a function of current density for SP/e-spunCL membranes and Nafion^®^115 membranes. The membrane morphology which is exfoliated structure effected power density. The highest power density 0.00118 Wcm^−2^ found in SP/e-spunCL15 which was observed morphologically exfoliated among all membranes. SP/e-spunCL25 (0.000710 Wcm^−2^) and (0.000772 Wcm^−2^) SP/e-spunCL20 smaller value of power density were observed in less exfoliated samples as compared to SP/e-spun17. Sp/e-spunCL10 and SP/e-spunCL30 with half-exfoliated structure, which resulted 0.000744 Wcm^−2^ and 0.000315 Wcm^−2^ respectively. Nevertheless, all SP/e-spunCL membranes observed higher power density than that of Nafion^®^ 115 with 0.000550 Wcm^−2^. The result has shown the potential of SP/e-spunCL membranes, notably SP/e-spunCL15, as alternatives for Nafion^®^ 115 due to better performance. However, the value obtained was rather lower than the standard test. The improvement in future should be studied not only on membranes but also optimization of the system such as methanol concentration, cooling system and MEA set up.

Beside the exfoliated structure desired for high power density PEMs, a good distribution also contributed to a high performance PEMs for DMFC as well. A good distribution causes a low ohmic resistance. Hence, in more oriented electrolyte, the proton from the anode side could transport more efficient to the cathode side and this would accelerate the kinetic of the reactions in both electrodes. This explains the essential of oriented nanostructures that contribute ion diffusion in the proper direction for the electrochemical processes occurring at each electrode. 

## 4. Conclusions

Eleccrospinning is a method that produces a well dispersed cloisite15A in SP/e-spunCL membranes. The SP/e-spunCL membranes with different degree of dispersions contributed to different morphologies. The exfoliated structure with the highest degree of cloisite15A dispersion found in SP/e-spunCL15 membrane. Performance test reported that the morphology together with good dispersion also improved membrane dimensional stability as well as DMFC performance. The performance results also showed that the power density of the SP/e-spunCL15 was higher as compared to Nafion^®^115 membrane. These features enable SP/e-spunCL15 membrane to be chosen as the most promising polymer electrolyte membrane that can be applied for DMFC. 

## Figures and Tables

**Table 1:** The designation of membrane samples.

Samples	Voltage (kV)	Distance (cm)	Designation
0.10 wt.% electrospun SPEEK/Cloisite membrane	22.5	20	SP/e-spunCL10
0.15 wt.% electrospun SPEEK/Cloisite membrane	22.5	20	SP/e-spunCL15
0.20 wt.% electrospun SPEEK/Cloisite membrane	22.5	20	SP/e-spunCL20
0.25 wt.% electrospun SPEEK/Cloisite membrane	22.5	20	SP/e-spunCL25
0.30 wt.% electrospun SPEEK/Cloisite membrane	22.5	20	SP/e-spunCL30

**Table 2:** Transmission bands of casted SPEEK/cloisite and SP/e-spunCL membranes.

Transmission Bands (cm^−1^)	Functional Groups
3400	O–H from sulfonic group
1640	C=O
1600	Aromatic C=C
1500	Aromatic C–C
1420	1,3,4-trisubstitured aromatic C–C skeletal vibrations
1210–1170	Asymmetric O=S=O stretching
1000	Symmetric O=S=O stretching
700	S–O stretch

**Table 3:** EDX quantitative analysis of dispersion of cloisite15A in SP/e-spunCL nanocomposite membranes.

Surface Elements	Surface Elements (wt.%)				
	SP/e-spunCLs10	SP/e-spunCL15	SP/e-spunCL20	SP/e-spunCL25	SP/e-spunCL30
C	40.94	41.19	41.01	41.42	42.88
O	21.69	21.94	21.76	22.76	24.63
Si	11.29	13.89	11.36	13.36	11.23
S	24.81	19.30	24.53	19.12	20.05
Mg	0.00	0.09	0.00	0.00	0.00
Al	0.00	0.07	0.00	0.00	0.00
Cu	1.27	3.52	1.34	3.34	1.21

**Table 4:** Mean of cloisite size in every10 µm^2^ SP/e-spunCL membranes.

Membrane Samples	Size of Closisite (µm)	Volume of Cloisite (µm^3^)	Number of Cloisite	Water Retained by Cloisite (µm^3^)
SP/e-spunCL10	0.286	7.318 × 10^−4^	1795	1.314
SP/e-spunCL15	0.301	2.914 × 10^−3^	1668	4.854
SP/e-spunCL20	0.357	1.472 × 10^−3^	1197	1.762
SP/e-spunCL25	0.304	1.681 × 10^−3^	1606	2.700
SP/e-spunCL30	0.390	4.505 × 10^−3^	1097	0.494

**Table 5:** Swelling properties of SPEEK and its corresponding nanocomposite membranes with various DS in water.

Sample	Weight Dry (mg)	Weight Wet (mg)	Thickness Dry (mm)	Thickness Wet (mm)	Diameter Dry (mm)	Diameter Wet (mm)	Percentage of Water Uptake and Dimensional Changes (%)		
							Water Uptake (%)	Swelling in Thickness (%)	Swelling in Plane (%)
SP/e-spunCL10	0.02 ± 0.65	0.026 ± 0.23	0.02 ± 0.65	0.022 ± 0.43	25.87 ± 0.54	25.9 ± 0.23	30	7.8	0.116
SP/e-spunCL15	0.01 ± 0.78	0.0126 ± 0.08	0.009 ± 0.06	0.011 ± 0.07	16.78 ± 0.65	16.8 ± 0.05	25.87	8.9	0.119
SP/e-spunCL20	0.03 ± 0.54	0.037 ± 0.76	0.024 ± 0.56	0.026 ± 0.05	17.876 ± 0.02	18 ± 0.06	24.56	8.33	0.694
SP/e-spunCL25	0.01 ± 0.64	0.012 ± 0.09	0.078 ± 0.21	0.163 ± 0.04	18.86 ± 0.97	19 ± 0.65	20	11	0.742
SP/e-spunCL30	0.01 ± 0.43	0.0119 ± 0.87	0.015 ± 0.32	0.018 ± 0.65	19.78 ± 0.34	20 ± 0.76	19	20	1.112

**Table 6:** Latest DMFC performances based on comparable materials and fabrications.

Reference Number	Materials	OCV (V)	Current (mAcm^−2^)	Power Density (Wcm^−2^)
[47]	PVDF/(PMMA-*co*-PAMPS)/SiO_2_	0.243	140.00	0.034.3
[48]	SPEEK (DS 67%)	1.006	672.00	0.191
[48]	SPEEK/CN-0.5	1.020	832.00	0.266
[49]	SPEEK-Sulfonation of fullerene (Sfu)	0.1	100.00	0.103
[50]	SPEEK-0.77% tetra(4-pyridyl)porphyrin (TPyP) (TPyP)	1.108	2.4 × 10^−4^	0.093
[51]	Electrospun phthalazinone ether sulfone ketone (SPPESK)/IEC1.72	0.92	350.00	1.000
-	Nafion^®^ 115	0.403	961.50	0.00055
-	SP/e-spunCL15	0.412	1042.20	0.00118
